# Agarwood Alcohol Extract Protects against Gastric Ulcer by Inhibiting Oxidation and Inflammation

**DOI:** 10.1155/2021/9944685

**Published:** 2021-09-18

**Authors:** Canhong Wang, Deqian Peng, Yangyang Liu, Yulan Wu, Peng Guo, Jianhe Wei

**Affiliations:** ^1^Hainan Provincial Key Laboratory of Resources Conservation and Development of Southern Medicine, Key Laboratory of State Administration of Traditional Chinese Medicine for Agarwood Sustainable Utilization, Hainan Branch Institute of Medicinal Plant Development, Chinese Academy of Medical Sciences and Peking Union Medical College, Haikou 570311, China; ^2^School of Pharmacy, Hainan Medical College, Haikou 571199, China; ^3^National Engineering Laboratory for Breeding of Endangered Medicinal Materials and Key Laboratory of Bioactive Substances and Resources Utilization of Chinese Herbal Medicine, Institute of Medicinal Plant Development, Chinese Academy of Medical Sciences & Peking Union Medical College, Beijing 100193, China

## Abstract

**Background:**

Agarwood has been used for centuries, especially for treatment of gastrointestinal diseases. Earlier studies of our laboratory suggested that agarwood alcohol extracts (AAEs) provided gastric mucosal protection. This study aims to investigate the ameliorative effect of AAEs on ethanol-induced gastric ulcers and its mechanism.

**Methods:**

Mice were given agarwood induced by the whole-tree agarwood-inducing technique alcohol extract (WTAAE, 0.71, 1.42, and 2.84 g/kg), wild agarwood induced by axe wounds alcohol extract (WAAE, 2.84 g/kg), and burning-chisel-drilling agarwood alcohol extract (FBAAE, 2.84 g/kg) orally, respectively. After 7 days' pretreatment with AAEs, the gastric ulcers were induced by absolute ethanol. The ulcer index, gastric histopathology, biochemical parameters, and inflammatory proteins were evaluated.

**Results:**

Pharmacological results showed AAEs (1.42 and 2.84 g/kg) reduced the gastric occurrence and ulcer inhibition rates up to more than 60%. AAEs decreased the level of nitric oxide (NO) and increased glutathione (GSH) and superoxide dismutase (SOD) levels. Besides, AAEs decreased the levels of interleukin-1*β* (IL-1*β*) and interleukin-6 (IL-6), but the interleukin-10 (IL-10) was upregulated. The expressions of nuclear factor kappa B (NF-*κ*B) and phosphorylated protein 38 (p-P38) were inhibited. The effect of WTAAE was better than that of FBAAE and similar to that of WAAE at the dose of 2.84 g/kg.

**Conclusions:**

These results demonstrate that agarwood alleviates the occurrence and development of gastric ulcers via inhibiting oxidation and inflammation.

## 1. Background

Agarwood, *Aquilaria* spp. (Thymelaeaceae), is a highly precious fragrant wood containing resin [1]. Agarwood is a famous traditional Chinese medicine that has been used for more than a thousand years for the treatment of various diseases; especially in the treatment of gastrointestinal diseases have better curative effect [2, 3]. It has been shown that sesquiterpenes and 2-(2-phenylethyl)chromone derivatives are two predominant constituents of agarwood [4, 5]. Our previous study showed that the components of WTAAE included sesquiterpenes (10.615%), chromone (31.678%), aromatics (31.831%), and other known compounds (25.760%) and also showed that sesquiterpenes and chromone are two predominant constituents of agarwood [6]. Agarwood were reported having anti-inflammatory properties, alleviating pain and other biological activities. These effects support its folkloric and clinical use for treating the painful and inflammatory diseases, such as gastric ulcers, gastritis, and angina [7, 8]. The extracts, essential oil, and main compounds from agarwood have exhibited extensive pharmacological activity properties, including sedative [9, 10], antineuroinflammatory [11], laxative [3], antioxidant [12, 13], anti-inflammatory [14, 15], and antibacterial [16] activities. Clinical application of TCM has found that agarwood have significant curative effects in the treatment of peptic ulcer, stomachache, and functional dyspepsia [17–19], but the effect and mechanism of treating gastrointestinal diseases are still unclear. Having research has been reported in vivo that agarwood water and alcohol solution can relieve intestinal tonic spasm in mice [20] and agarwood extract also protects against gastric ulcer in rats [21]. So, we speculated that agarwood may play an antigastric ulcer role by improving gastrointestinal motility and anti-inflammation. Our previous in vivo study also confirmed that WTAAE provided gastric mucosal protection and inhibited the occurrence of gastric ulcer in ethanol-induced rats [22]. However, the mechanism of WTAAE in preventing the formation of gastric ulcers has not been verified by pharmacological studies.

Gastric ulcer is a common gastrointestinal disease and affects approximately 5–10% of people, which occurrence and development are the complex process [23]. The main risk factors that cause gastric ulcers include alcohol, smoking, nonsteroidal anti-inflammatory drugs, etc. [24]. Excessive alcohol increased mucosal permeability, mucosal damage, cell necrosis, and inflammatory responses [25, 26].

The mechanisms of gastric injury have not been fully clarified, but it is well known that proinflammatory mediators played an important role in the development of ulcer, including IL-6, IL-10, tumor necrosis factor-*α* (TNF-*α*), and NF-*κ*B [27]. Furthermore, NO was a major defensive system in the gastric mucosa [28]. At present, the synthetic drugs have high efficacy for gastric ulcer therapy such as PPIs. However, their prolonged use may cause side effects [29, 30]. Therefore, one of our purposes is to search the nontoxic natural drugs that can prevent and treat gastric ulcers.

This study aims to investigate the effect of WTAAE on ethanol-induced gastric ulcers and explore the potential mechanisms. Furthermore, we compare the protective effect of different AAEs against gastric ulcers. The research provides reference evidence for further study into agarwood's protective mechanism of action against gastrointestinal inflammatory diseases.

## 2. Methods

### 2.1. Reagents

The biochemical kits NO, GSH, SOD, and BCA protein assay were provided by the Institute of Jiancheng Biotech Co. (Nanjing, China). The enzyme-linked immunosorbent assay (ELISA) kits for determination of cytokines (IL-1*β*, IL-6, and IL-10) were produced by Bossbio Biotech (Beijing, China). NF-*κ*B and p-P38 were purchased from Santa Cruz Biotech (Santa Cruz, CA, USA). Anhydrous ethanol and other chemicals procured were of analytical grade.

### 2.2. Preparation of the Extract from Agarwood

The agarwood (specimen number: JC2016112) produced by whole-tree agarwood-inducing technique (patent number: ZL201010104119.5), wild agarwood (specimen number: JC2016099) induced by axe wounds, and burning-chisel-drilling agarwood (specimen number: JC2016076) were purchased from Guangdong in China. They were identified by Professor Jianhe Wei (Institute of Medicinal Plant Development, Chinese Academy of Medical Sciences & Peking Union Medical College, Beijing, China). Agarwood (1000 g) was dried, smashed, and soaked with 95% ethanol (5 L) for 2 h. The resulting mixture was extracted by reflux extraction for 1 h and filtered. The procedure was repeated two times. The resulting ethanol solutions were combined, concentrated, and dried in vacuo to obtain dark brown WTAAE (140 g, 14%), which was stored in a freezer at −20°C. The ethanol extracts of WAAE and FBAAE were produced by the same method (the extraction rates of 10.5% and 14%, respectively).

### 2.3. Animals and Experimental Procedure

Male ICR mice (20 ± 2 g, 4–6 weeks) were purchased from Vital River Laboratory Animal Technology Co., Ltd. (Beijing, China). Animals were kept in a 12-h light/dark cycle at a temperature of 20–24°C and humidity of 50–70% with access to food and water ad libitum and were housed for three days prior to experiments. Animal care and experimental protocols were approved by the Animal Care and Use Committee at the Institute of Medicinal Plant Development, Chinese Academy of Medical Sciences.

The seventy mice were randomly divided into seven groups. The groups were as follows: normal control group, model group, WAAE 2.84 group, FBAAE group, and three WTAAE groups. The normal and model groups were orally administered with 20 mL/kg of distilled water. The WAAE and FBAAE groups were pretreated with 2.84 g/kg daily by oral garage. The WTAAEs were administered with 0.71, 1.42, and 2.84 g/kg of agarwood extracts, respectively. After pretreatment for 7 days and being deprived of food for 24 h with water ad libitum, other mice were infected and the mouse acute gastric ulcer model was made by oral gavage absolute ethanol at a concentration of 0.15 mL/10 g except for the normal group. 1 h after administration with absolute ethanol, whole blood samples were collected from the orbit for serum analysis, and animals were sacrificed by cervical dislocation. Then, the stomachs were immediately removed and fixed with formaldehyde solution and were prepared for examination of the gastric lesion index and histopathologic sections.

### 2.4. Determination of Ulcer Index

The stomachs were fixed in 4% formaldehyde solution for 1 h. We observed the degree of gastric mucosal defects both the inner and outer layer. The length (mm) of each mucosal lesion was measured with visual inspection. The score was estimated as follows: the blood point is 1 score, the line length is less than 1 mm for 2 scores, 1–2 mm for 3 scores, 3–4 mm for 4 scores, and 5 mm and longer for 5 scores [31]. The total scores of the whole stomach amounted to the ulcer index. We also calculated the ulcer inhibition rate. The percentage ulcer inhibition rate was calculated as follows: ulcer inhibition rate (%) = (mean ulcer indexes of model group − mean ulcer indexes of treatment group)/mean ulcer indexes of model group × 100.

### 2.5. Tissue Damage Evaluation

Stomach tissues were fixed in formaldehyde solution, dehydrated, embedded in paraffin, and sectioned. For tissue damage analysis, the 5 *µ*m sections were stained with hematoxylin-eosin (HE) using standard operation, observed under a microscope at the magnification of 400×, and photographed. Tissue damage was calculated using the following score system (LDI score): a 1 cm segment of each tissue damage section was assessed for epithelial cell loss (score: 0–3), edema in the upper mucosa (score: 0–4), hemorrhagic damage (score: 0–4), and the presence of inflammatory cells (score: 0–3) [32].

### 2.6. Assessment of Oxidative Stress in Tissues

The stomach tissues were fully homogenized with ice-cold saline (1 : 9 w/v) using a homogenizer. After being centrifuged at 3000 rpm at 4°C for 15 min, the stomach tissue supernatant was collected and used for assaying the contents and enzymes of oxidative indexes. The levels of NO, GSH, and SOD were measured using commercial kits following the manufacturer's instructions.

### 2.7. Detection of IL-1*β*, IL-6, and IL-10 in Serum

Serum samples were collected to assess the effects of agarwood extracts on mice stomach tissue. The cytokines of IL-1*β*, IL-6, and IL-10 in serum were measured using specific mouse ELISA kits according to the manual instructions.

### 2.8. Immunohistochemical (IHC) Analysis of Stomach Tissue

The stomach tissues were fixed in formalin solution, dehydrated with ethanol, embedded in paraffin, and sectioned. The 5 *µ*m sections were sealed with a buffered blocking solution in phosphate-buffered saline (PBS) (containing 3% bovine serum albumin) for 15 min. Then, the sections were coincubated with the primary antibody for NF-*κ*B and p-P38 at a dilution of 1 : 50 in PBS (v/v) at 4°C overnight. Then, the sections were washed with PBS and incubated with the secondary antibody at a dilution of 1 : 500 in PBS (v/v) at room temperature for 1 h. Thereafter, sections were washed with 0.05 M of Tris-HCl at a pH of 7.66 and coincubated with a 3,3'-diaminobenzidine solution in darkness at room temperature for 10 min. The sections were washed again, stained with hematoxylin, and observed under a light microscope. The Image-ProPlus 6.0 software (Media Cybernetics, Rockville, MD, USA) was used to analyze the expression of proteins.

### 2.9. Statistical Analysis

Data were expressed as mean ± SD for ten animals in each group and were statistically evaluated with SPSS 17.0 software (IBM Corporation, New York, NY, USA). Differences between each group were analyzed by one-way ANOVA and Tukey's post hoc test (TTEST). Results with *p* < 0.05 were considered statistically significant.

## 3. Results

### 3.1. Effect of AAEs on Gastric Lesions and Ulcer Inhibition Rates

**Figure 1:**
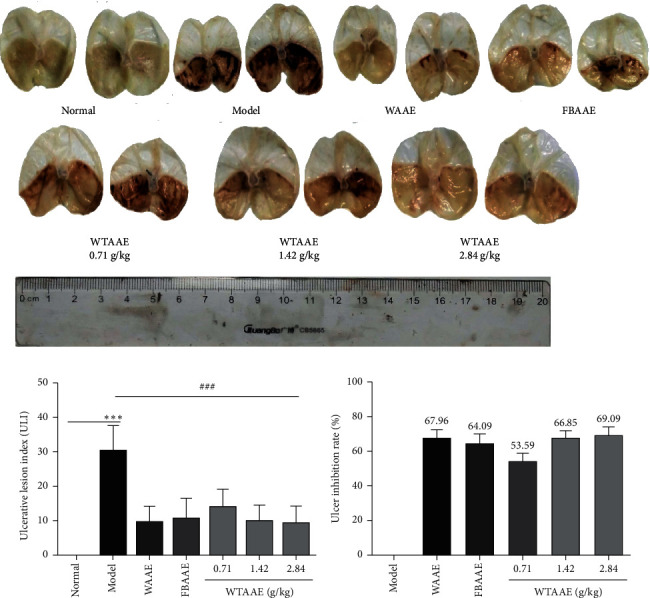
Effect of AAEs on gastric ulcer induced by anhydrous ethanol in mice. Animals were treated with WAAE (2.84 g/kg), FBAAE (2.84 g/kg), or WTAAE (0.71, 1.42, and 2.84 g/kg) by oral administration. The normal and model groups were given distilled water correspondingly. (a) Typical pictures of stomachs. (b) Gastric lesions index. (c) Ulcer inhibition rate. Data are expressed as mean ± SD (*n* = 6). ^*∗∗∗*^*p* < 0.001 vs. normal group; ^###^*p* < 0.001 vs. model group.

**Figure 2:**
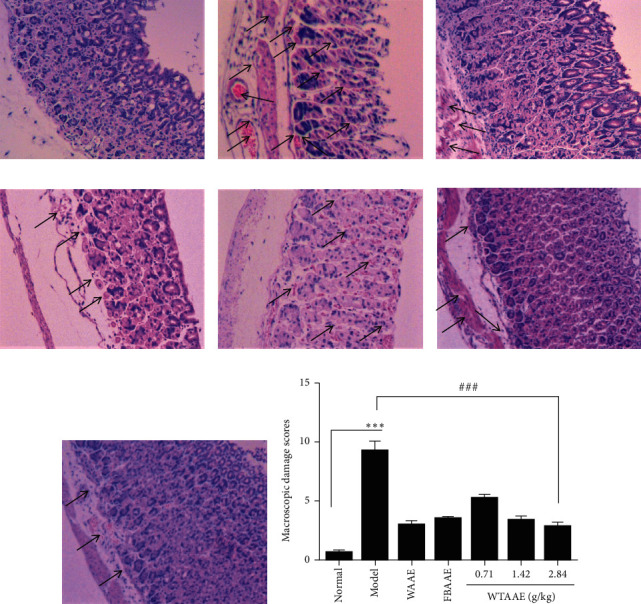
Effect of the AAEs on gastric histopathology stained by hematoxylin and eosin (H&E). Animals were treated with WAAE (2.84 g/kg), FBAAE (2.84 g/kg), or WTAAE (0.71, 1.42, and 2.84 g/kg) by oral administration. The normal and model groups were given distilled water correspondingly. (a) Normal. (b) Model. (c) WAAE (2.84 g/kg). (d) FBAAE (2.84 g/kg). (e) WTAAE (0.71 g/kg). (f) WTAAE (1.42 g/kg). (g) WTAAE (2.84 g/kg). (h) Pathological damage score analysis from HE staining. Data are expressed as mean ± SD (*n* = 4). ^*∗∗∗*^*p* < 0.001 vs. normal group; ^###^*p* < 0.001 vs. model group.

Gastric lesions were determined by measuring the ulcer index on the gastric mucosal surface. As shown in Figure 1(a), severe gastric ulcerations were induced by anhydrous ethanol in mice with the ulcer index being significantly increased (30.17 ± 7.28), characterized by linear hemorrhages and ulceration craters in the mucosal layer. Fortunately, pretreatment with AAEs reduced ethanol-induced mucosal damage with the ulcer index, as shown in Figure 1(b). The ulcer index of WAAE was 9.67 ± 4.63, FBAAE was 10.83 ± 5.81, and WTAAE was 9.33 ± 5.05 with a dose of 2.84 g/kg; *p* < 0.001 compared with the model group, respectively. The ulcer inhibition rate showed the protective effect of AAEs against gastric ulcers, as in Figure 2(c). The ulcer inhibition rates were enhanced in a dose-dependent manner for WTAAE groups (53.59, 66.85, and 69.09%), WAAE (67.96%), and FBAAE (64.09%) groups. Similar inhibition rates were found with the same doses of WTAAE and WAAE, which were better than the FBAAE group. These results showed that AAEs had a significant effect on inhibiting the occurrence of gastric ulcers.

### 3.2. Effect of AAEs on Tissue Damage

Administration of anhydrous ethanol induced significantly tissue damage changes including the appearance of submucosa edema, hemorrhagic injury, mucosa degradation, epithelial cell loss, inflammatory cell infiltration, and necrosis (Figure 2(b)). Pretreatment with AAEs considerably reduced these changes in the gastric mucosa and provided protection against gastric lesions. Mice administered with WAAE and FBAAE presented a small swelling caused by anhydrous ethanol, with lower numbers of inflammatory cells (Figures 2(c) and 2(d)). The gastric mucosa of mice treatment with 0.71, 1.42, and 2.84 g/kg of WTAAE showed a dose-dependent protection (Figures 2(e)–2(g)). The quantitative analysis diagram shows this result directly (Figure 2(h)). Furthermore, the WTAAE and WAAE of 2.84 g/kg dose had a similar effect, which is more potent than the effect of FBAAE.

### 3.3. Antioxidative Stress Activity of AAEs

**Figure 3:**
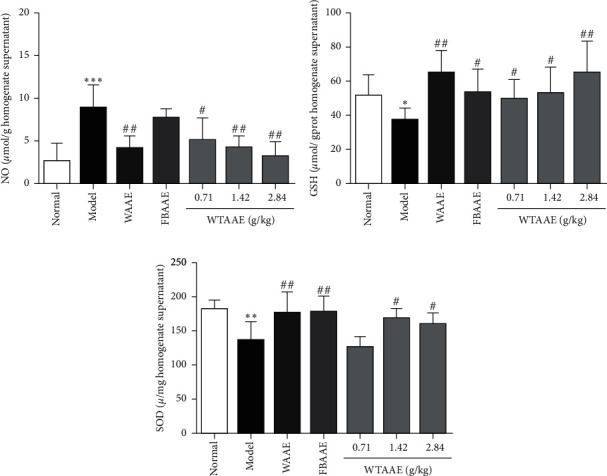
Effect of the AAEs on the levels of NO, GSH, and SOD in homogenate supernatant of stomach tissues. Animals were treated with WAAE (2.84 g/kg), FBAAE (2.84 g/kg), or WTAAE (0.71, 1.42, and 2.84 g/kg) by oral administration. The normal and model groups were given distilled water correspondingly. Data are expressed as mean ± SD (*n* = 6). ^*∗*^*p* < 0.05, ^*∗∗*^*p* < 0.01, ^*∗∗∗*^*p* < 0.001 vs. normal group; ^#^*p* < 0.05, ^##^*p* < 0.01 vs. model group.

Compared with the normal group, the model group showed significant oxidative changes with increasing lipid peroxidation, such as the increase of NO levels (8.94 ± 2.63; *p* < 0.001; Figure 3(a)). Interestingly, AAEs protected against lipid peroxidation in the ulcer tissues. The NO levels were obviously decreased in the WAAE (4.08 ± 1.49) and WTAAE (3.22 ± 1.70) groups of 2.84 g/kg, especially the WTAAE groups with the doses of 0.71, 1.42, and 2.84 g/kg showing a dose-dependent reduction (*p* < 0.05 or *p* < 0.01). GSH content (37.53 ± 6.64) and SOD activity (137.01 ± 26.05) were significantly decreased in the model group (Figures 3(b) and 3(c)) but were increased in the groups provided with AAE pretreatment (*p* < 0.05 or *p* < 0.01). These results suggested that AAEs have obvious antioxidant effect, and the effect of WTAAE was similar to that of WAAE at the same dose.

### 3.4. Anti-Inflammatory Cytokines Production of AAEs

**Figure 4:**
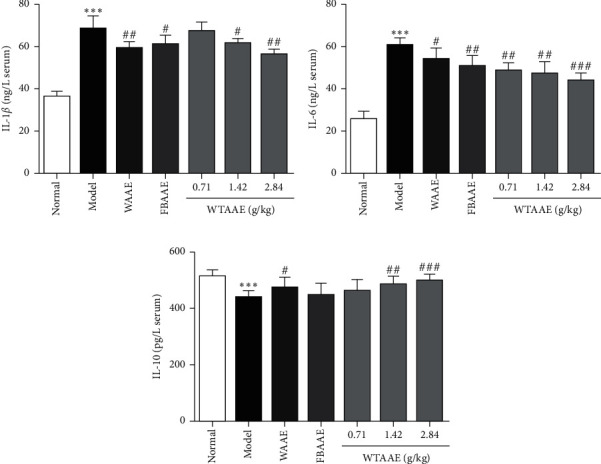
Effect of the AAEs on the levels of IL-1*β*, IL-6, and IL-10 in mice serum. Animals were treated with WAAE (2.84 g/kg), FBAAE (2.84 g/kg), or WTAAE (0.71, 1.42, and 2.84 g/kg) by oral administration. The normal and model groups were given distilled water correspondingly. Data are expressed as mean ± SD (*n* = 6). ^*∗∗∗*^*p* < 0.001 vs. normal group; ^#^*p* < 0.5, ^##^*p* < 0.01, ^###^*p* < 0.001 vs. model group.

To evaluate the anti-inflammation of the AAEs in ulcers, we detected the levels of proinflammatory and anti-inflammatory cytokines, including IL-1*β*, IL-6, and IL-10 in the serum. As shown in Figures 4(a)–4(c), the levels of proinflammatory cytokines IL-1*β* (68.77 ± 5.74) and IL-6 (60.51 ± 3.49) were significantly increased in the gastric ulcer mice (*p* < 0.001) compared with the normal group. AAE treatment dramatically attenuated the anhydrous ethanol-induced elevation of these proinflammatory cytokines (the IL-1*β* level of WAAE was 59.23 ± 3.07, FBAAE was 61.38 ± 4.06, and WTAAE was 56.30 ± 2.32, together with the IL-6 level of WAAE was 54.24 ± 4.95, FBAAE was 50.68 ± 5.19, and WTAAE was 43.99 ± 3.28 at the dose of 2.84 g/kg, *p* < 0.05 or *p* < 0.01). Particularly, the WTAAE groups showed the successive decrease of the proinflammatory cytokines, with the 2.84 g/kg dose having the lowest level. In contrast, the level of IL-10 (442.79 ± 21.75) was significantly decreased in the model group (*p* < 0.001), which was obviously increased in the WAAE (478.44 ± 35.74) and WTAAE (502.19 ± 19.62) groups at 2.84 g/kg, especially in 2.84 g/kg dose of the WTAAE group.

### 3.5. AAEs Inhibit the Expression of NF-*κ*B and p-P38

**Figure 5:**
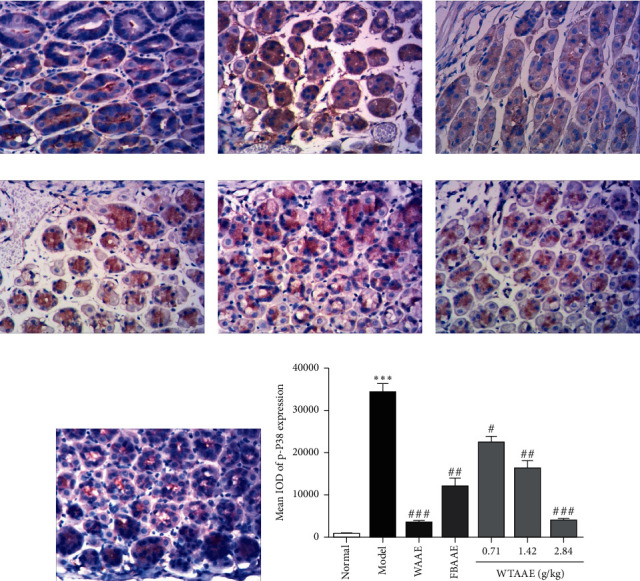
Effect of the AAEs on the protein expression of NF-*κ*B. Animals were treated with WAAE (2.84 g/kg), FBAAE (2.84 g/kg), or WTAAE (0.71, 1.42, and 2.84 g/kg) by oral administration. The normal and model groups were given distilled water correspondingly. (a) Normal. (b) Model. (c) WAAE (2.84 g/kg). (d) FBAAE (2.84 g/kg). (e) WTAAE (0.71 g/kg). (f) WTAAE (1.42 g/kg). (g) WTAAE (2.84 g/kg). (h) Pathological damage score analysis from HE staining and quantification of NF-*κ*B expression. Data are expressed as mean ± SD (*n* = 3). ^*∗∗∗*^*p* < 0.001 vs. normal group; ^#^*p* < 0.5, ^##^*p* < 0.01, ^###^*p* < 0.001 vs. model group.

**Figure 6:**
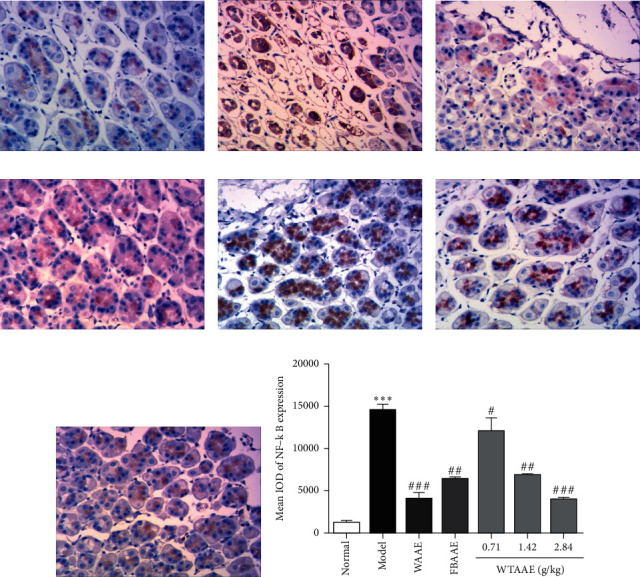
Effect of the AAEs on the protein expression of p-P38. Animals were treated with WAAE (2.84 g/kg), FBAAE (2.84 g/kg), or WTAAE (0.71, 1.42, and 2.84 g/kg) by oral administration. The normal and model groups were given distilled water correspondingly. (a) Normal. (b) Model. (c) WAAE (2.84 g/kg). (d) FBAAE (2.84 g/kg). (e) WTAAE (0.71 g/kg). (f) WTAAE (1.42 g/kg). (g) WTAAE (2.84 g/kg). (h) Pathological damage score analysis from HE staining and quantification of p-P38 expression. Data are expressed as mean ± SD (*n* = 3). ^*∗∗∗*^*p* < 0.001 vs. normal group; ^#^*p* < 0.5, ^##^*p* < 0.01, ^###^*p* < 0.001 vs. model group.

To further investigate the underlying anti-inflammatory molecular mechanisms of AAEs, we performed IHC to detect the levels of NF-*κ*B (Figure 5) and p-P38 (Figure 6) in the stomach tissue of gastric ulcer mice. The results showed that the level of NF-*κ*B was significantly increased in gastric ulcer mice of the model group (Figure 5(b)), but AAE pretreatment inhibited the NF-*κ*B phosphorylated expression (Figures 5(c)–5(g)). Similarly, we also found that phosphorylated p38 MAPK was significantly upregulated in the model group (*p* < 0.01; Figure 6(b)), while the levels of p-P38 were attenuated by the AAE pretreatment (*p* < 0.01; Figure 6(c)–6(g)). Furthermore, the quantitative analysis diagrams showed more obvious results (Figures 5(h) and 6(h)). These results suggested that AAEs evidently exert anti-inflammatory effects, with WTAAE's effect being similar to WAAE's and better than FBAAE's.

## 4. Discussion

Agarwood, a traditional Chinese medicine, had been widely used for treating various diseases for centuries, especially treatment of gastric ulcers. As a result of overexploitation on agarwood, natural products have been on the verge of extinction, which posed a serious threat to species diversity and medication safety. Fortunately, artificial agarwood-inducing methods by our research team could produce large quantities of high-quality agarwood, which reversed the condition and also made a basis on agarwood pharmacological investigation [1]. Our previous study has revealed that the agarwood extracts provided gastric mucosal protection [22]. The study first found that the gastroprotective effect of AAEs on ethanol-induced gastric injury was relieving the tissue damage degree of gastric tissue, decreasing the lipid peroxide production and the secretion of the inflammatory cytokines. Besides, this finding indicated that the effect of WTAAE was similar to that of WAAE, which was better than that of FBAAE in improving the occurrence and development of gastric ulcer. Furthermore, this study explained that a large amount of high-quality agarwood produced by the whole-tree agarwood-inducing technique could be safely and effectively applied in the clinic of traditional Chinese medicine (TMC). Alcohol consumption is a common way to establish the gastric ulcer model. The result of gastric ulcer is characterized by mucosal edema, hemorrhages, and inflammatory cell infiltration [33, 34]. The appearance of these may be related to free radicals produced and severe hemorrhage [35]. All these ulcer indexes and histological lesions could be used to measure gastric mucosal damage. Our study showed that administration of anhydrous ethanol (0.15 mL/10 g) produced noticeable mucosal damage. Pretreatment with AAEs substantially reduced the ulcer index and the areas of gastric ulcer histological lesion formation compared with ulcer of the model group (Figures 1 and 2).

NO plays a crucial role in various physiological processes, such as dilating blood vessels and stimulating gastric angiogenesis of ulcers. Some studies have demonstrated that the endogenous NO provides protection for the gastric mucosa [36, 37]. However, the excessive NO could also stimulate cell proliferation of the gastric mucosa and granulation tissue formation [38]. GSH and SOD are important antioxidants, which protect the gastric mucosa from oxidative damage [39], as they could scavenge free radicals. Therefore, the lower levels of GSH and SOD also indicate the severity of oxidative stress damage [37, 40]. With the gastric mucosa damaged by ethanol, GSH, SOD, and GSH-Px decreased significantly, while NO and MDA increased [41]. Our study confirmed increasing NO and decreasing GSH and SOD in the model group, which suggests that gastric mucosal damage was achieved by ethanol. Furthermore, our results also showed that AAEs had antioxidant effects and protected against gastric injuries by scavenging free radicals, which could decrease the NO level in addition to increase of the GSH content and SOD activity. In addition, our previous study found that WTAAE could play a better antimyocardial ischemia effect through antioxidant effect, and it also proved that it could improve the tissue damage caused by different diseases through antioxidant effect [42].

Previous research showed that gastric ulcers caused by ethanol may activate the innate immune system and upregulate the levels of proinflammatory cytokines, such as TNF-*α*, IL-1*β*, and IL-6 [43]. Furthermore, IL-10 played an important role in downregulating the inflammatory pathway and preventing autoimmune pathologies [37, 44, 45]. Our results suggested that AAEs has potential anti-inflammatory effects on the ethanol-induced gastric damage. Pretreatment with AAEs significantly reduced the proinflammatory cytokine levels of IL-1*β* and IL-6 and enhanced the anti-inflammatory cytokine IL-10 in the gastric ulcer mice, suggesting that they provide protection against the inflammation of gastric ulcer.

Inflammation is another crucial factor involved in the pathogenesis of gastric ulcer. It is well known that NF-*κ*B is a vital transcription factor in the acute-phase inflammatory processes, which could induce upregulation of the proinflammatory cytokines TNF-*α*, IL-6, and IL-1*β* [43]. When gastric mucosa cells stimulated by exogenous stimuli containing ethanol, NF-*κ*B was activated and combined with specific gene-starting factors, which triggered the target inflammatory cytokines and aggravated gastric mucosal damage [37, 46]. The results of this study suggested that pretreatment with AAEs inhibited NF-*κ*B activation and decreased the expression of NF-*κ*B, which could possibly contribute to alleviating the inflammatory injury of gastric ulcer. The effect of WTAAE was better than that of FBAAE and similar to that of WAAE. Besides, the decrease of NF-*κ*B also reduced the secretion of proinflammatory cytokines, which regulated the inflammation of gastric ulcers using negative feedback. Besides, our previous study found that WTAAE could mitigate intestinal injury through upregulation of the Nrf2-ARE pathway and downregulation of the NF-*κ*B pathway, and it also proved that it could improve the tissue damage caused by different diseases through antioxidant and anti-inflammatory effects [37].

p38 is one of the signaling pathways of mitogen-activated protein kinases (MAPKs) and plays an important role in the regulation of various toxic substance-induced damage and repairs the processes of gastric mucosa epithelium [47]. It is well known that the activation of p38 MAPK was transferred into the nucleus and regulated the expressions of inflammation-related genes [48]. The results of this study showed that AAEs also significantly decreased the expression of p38 protein phosphorylation and inhibited inflammation. Therefore, we believed that AAEs could reduce ethanol-induced gastric ulcers and promote the repair of gastric mucosal damage.

## 5. Conclusions

The study demonstrated that AAEs exerted protective effects against the ethanol-induced gastric ulcers, with the effects of WTAAE found to be better than that of FBAAE and similar to that of WAAE. Their potential mechanisms related to antioxidative and anti-inflammatory activities. Our results have provided experimental evidence for further research and the clinical application of agarwood against gastric ulcers. However, the pharmacodynamic substances and specific mechanism of agarwood against gastric ulcer need to be further investigated and confirmed. To sum up, the study suggested that agarwood might be useful in the prevention and treatment of gastrointestinal diseases.

## Data Availability

Materials used and data collected in this study are available from the first author on reasonable request.

## Abbreviations

AAEs: Agarwood alcohol extracts
ELISA: Enzyme-linked immunosorbent assay
FBAAE: Burning-chisel-drilling agarwood alcohol extract
GSH: Glutathione
IL-1*β*: Interleukin-1*β*
IL-6: Interleukin-6
IL-10: Interleukin-10
IHC: Immunohistochemical
HE: Hematoxylin-eosin
MAPK: Mitogen-activated protein kinase
NF-*κ*B: Nuclear factor kappa B
NO: Nitric oxide
PBS: Phosphate-buffered saline
p-P38: Phosphorylated protein 38
SOD: Superoxide dismutase
TMC: Traditional Chinese medicine
TNF-*α*: Tumor necrosis factor-*α*
WTAAE: Whole-tree agarwood-inducing technique alcohol extract
WAAE: Wild agarwood induced by axe wounds alcohol extract.

## Supplementary Materials

Supplementary Materials Supplemental Files1. Graphical abstract. Supplemental Files2. Highlights. Supplemental Files3. Chromatogram of chemical components of WTAAE. Supplemental Files4. Table 1 Chemical compositions and relative amounts of the WTAAE.

## References

[1] Liu Y., Chen H., Yang Y. (2013). Whole-tree agarwood-inducing technique: an efficient novel technique for producing high-quality agarwood in cultivated Aquilaria sinensis trees. *Molecules*.

[2] Borris R. P., Blaskó G., Cordell G. A. (1988). Ethnopharmacologic and phytochemical studies of the Thymelaeaceae. *Journal of Ethnopharmacology*.

[3] Kakino M., Izuta H., Ito T. (2010). Agarwood induced laxative EffectsviaAcetylcholine receptors on loperamide-induced constipation in mice. *Bioscience, Biotechnology, and Biochemistry*.

[4] Chen H.-Q., Wei J.-H., Yang J.-S. (2012). Chemical constituents of agarwood originating from the endemic genus Aquilaria plants. *Chemistry and Biodiversity*.

[5] Li W., Cai C.-H., Dong W.-H. (2014). 2-(2-Phenylethyl)chromone derivatives from Chinese agarwood induced by artificial holing. *Fitoterapia*.

[6] Wang C. H., Wang S., Peng D. Q., Yu Z. X., Guo P., Wei J. H. (2018). Protective effect of agarwood alcohol extracts produced by whole-tree agarwood-inducing technique on the Fluorouracil -induced liver injury in mice. *International Journal of Pharmaceutical Research*.

[7] Zhou M., Wang H., Suolangjiba J., Kou J., Yu B. (2008). Antinociceptive and anti-inflammatory activities of Aquilaria sinensis (Lour.) Gilg. Leaves extract. *Journal of Ethnopharmacology*.

[8] Zhu Z., Zhao Y., Huo H. (2016). Hhx-5, a derivative of sesquiterpene from Chinese agarwood, suppresses innate and adaptive immunity via inhibiting stat signaling pathways. *European Journal of Pharmacology*.

[9] Okugawa H., Ueda R., Matsumoto K., Kawanishi K., Kato A. (1993). Effects of agarwood extracts on the central nervous system in mice. *Planta Medica*.

[10] Wang S., Wang C., Peng D. (2017). Agarwood essential oil displays sedative-hypnotic effects through the GABAergic system. *Molecules*.

[11] Huo H.-X., Zhu Z.-X., Pang D.-R. (2015). Anti-neuroinflammatory sesquiterpenes from Chinese eaglewood. *Fitoterapia*.

[12] Yang X.-B., Feng J., Yang X.-W., Zhao B., Liu J.-X. (2012). Aquisiflavoside, a new nitric oxide production inhibitor from the leaves of Aquilaria sinensis. *Journal of Asian Natural Products Research*.

[13] Sattayasai J., Bantadkit J., Aromdee C., Lattmann E., Airarat W. (2012). Antipyretic, analgesic and anti-oxidative activities of Aquilaria crassna leaves extract in rodents. *Journal of Ayurveda and Integrative Medicine*.

[14] Zhu Z., Gu Y., Zhao Y., Song Y., Li J., Tu P. (2016). Gyf-17, a chloride substituted 2-(2-phenethyl)-chromone, suppresses LPS-induced inflammatory mediator production in RAW264.7 cells by inhibiting STAT1/3 and ERK1/2 signaling pathways. *International Immunopharmacology*.

[15] Huo H.-X., Gu Y.-F., Sun H. (2017). Anti-inflammatory 2-(2-phenylethyl)chromone derivatives from Chinese agarwood. *Fitoterapia*.

[16] Kamonwannasit S., Nantapong N., Kumkrai P., Luecha P., Kupittayanant S., Chudapongse N. (2013). Antibacterial activity of Aquilaria crassna leaf extract against Staphylococcus epidermidis by disruption of cell wall. *Annals of Clinical Microbiology and Antimicrobials*.

[17] Chen S. S., Guan C. (2001). Treatment of 103 cases of stomachache with agarwood zhitong powder. *Jilin Academy of Traditional Chinese Medicine*.

[18] Maupin (2009). Clinical observation on 65 cases of peptic ulcer treated with modified agarwood powder. *Yunnan Journal of Traditional Chinese Medicine*.

[19] Cai Z. Z., Wang J. Z., Cao S. G. (2010). Therapeutic effect of Agarwood Hua-Qi Capsule on intestinal gas in patients with functional dyspepsia. *International Journal of Applied and Basic Medical Research*.

[20] Zhou Y. P. (1988). Pharmacological effects of agarwood on intestinal smooth muscle. *Journal of Traditional Chinese Medical Sciences*.

[21] Ma L., Zhang X., Chen H. X. (2019). Protective effect and mechanism of agarwood extract on ethanol-induced gastric ulcer in rats. *Herald of Medicine*.

[22] Liu Y. Y., Wang S., Zhou Y. (2016). Effect of agarwood extracts produced by the whole-tree agarwood-inducing technique on gastrointestinal motility and gastric ulcer. *International Journal of Pharmaceutical Research*.

[23] Sumbul S., Ahmad M. A., Mohd A., Mohd A. (2011). Role of phenolic compounds in peptic ulcer. *Journal of Pharmaceutical and Bioanalytical Science*.

[24] Dovjak P. (2017). Ulcus duodeni, Ulcus ventriculi und *Helicobacter pylori*. *Zeitschrift für Gerontologie und Geriatrie*.

[25] Yang Y., Wang Z., Zhang L. (2018). Protective effect of gentiopicroside from Gentiana macrophylla Pall. in ethanol-induced gastric mucosal injury in mice. *Phytotherapy Research*.

[26] Etani R., Kataoka T., Kanzaki N. (2017). Protective effects of hot spring water drinking and radon inhalation on ethanol-induced gastric mucosal injury in mice. *Journal of Radiation Research*.

[27] Ren S., Wei Y., Wang R. (2020). Rutaecarpine ameliorates ethanol-induced gastric mucosal injury in mice by modulating genes related to inflammation, oxidative stress and apoptosis. *Frontiers in Pharmacology*.

[28] Dambrova M., Zvejniece L., Skapare E. (2010). The anti-inflammatory and antinociceptive effects of NF-*κ*B inhibitory guanidine derivative ME10092. *International Immunopharmacology*.

[29] Bandyopadhyay D., Biswas K., Bhattacharyya M., Reiter R. J., Banerjee R. K. (2002). Involvement of reactive oxygen species in gastric ulceration: protection by melatonin. *Indian Journal of Experimental Biology*.

[30] de Lira Mota K. S., Dias G. E. N., Pinto M. E. F. (2009). Flavonoids with gastroprotective activity. *Molecules*.

[31] Amirshahrokhi K., Khalili A.-R. (2015). The effect of thalidomide on ethanol-induced gastric mucosal damage in mice: involvement of inflammatory cytokines and nitric oxide. *Chemico-Biological Interactions*.

[32] Laine L., Weinstein W. M. (1988). Histology of alcoholic hemorrhagic “gastritis”: a prospective evaluation. *Gastroenterology*.

[33] Ko J. K.-S., Cho C. H., Lam S. K. (2004). Adaptive cytoprotection through modulation of nitric oxide in ethanol-evoked gastritis. *World Journal of Gastroenterology*.

[34] Li C.-Y., Xu H.-D., Zhao B.-T., Chang H.-I., Rhee H.-I. (2008). Gastroprotective effect of cyanidin 3-glucoside on ethanol-induced gastric lesions in rats. *Alcohol*.

[35] Bharti S., Wahane V. D., Kumar V. L. (2010). Protective effect of Calotropis procera latex extracts on experimentally induced gastric ulcers in rat. *Journal of Ethnopharmacology*.

[36] Kim H., Kim K. H. (1998). Effect of nitric oxide on hydrogen peroxide-induced damage in isolated rabbit gastric glands. *Pharmacology*.

[37] Wang C., Wang S., Peng D., Yu Z., Guo P., Wei J. (2019). Agarwood extract mitigates intestinal injury in fluorouracil-induced mice. *Biological and Pharmaceutical Bulletin*.

[38] Li Y., Wang W. P., Wang H. Y., Cho C. H. (2000). Intragastric administration of heparin enhances gastric ulcer healing through a nitric oxide-dependent mechanism in rats. *European Journal of Pharmacology*.

[39] Kwiecien S., Brzozowski T., Konturek S. J. (2002). Effects of reactive oxygen species action on gastric mucosa in various models of mucosal injury. *Journal of Physiology and Pharmacology: An Official Journal of the Polish Physiological Society*.

[40] Kahraman A., Erkasap N., Köken T., Serteser M., Aktepe F., Erkasap S. (2003). The antioxidative and antihistaminic properties of quercetin in ethanol-induced gastric lesions. *Toxicology*.

[41] Rahman K. (2007). Studies on free radicals, antioxidants, and co-factors. *Clinical Interventions in Aging*.

[42] Wang C., Peng D., Liu Y., Yu Z., Guo P., Wei J. (2020). Agarwood alcohol extract ameliorates isoproterenol-induced myocardial ischemia by inhibiting oxidation and apoptosis. *Cardiology Research and Practice*.

[43] Sinha K., Sadhukhan P., Saha S., Pal P. B., Sil P. C. (2015). Morin protects gastric mucosa from nonsteroidal anti-inflammatory drug, indomethacin induced inflammatory damage and apoptosis by modulating NF-*κ*B pathway. *Biochimica et Biophysica Acta (BBA) - General Subjects*.

[44] Stordeur P., Goldman M. (1998). Interleukin-10 as a regulatory cytokine induced by cellular stress: molecular aspects. *International Reviews of Immunology*.

[45] Saraiva M., O’Garra A. (2010). The regulation of IL-10 production by immune cells. *Nature Reviews Immunology*.

[46] Gambhir S., Vyas D., Hollis M., Aekka A., Vyas A. (2015). Nuclear factor kappa B role in inflammation associated gastrointestinal malignancies. *World Journal of Gastroenterology*.

[47] Wu H.-L., Gao X., Jiang Z.-D. (2013). Attenuated expression of the tight junction proteins is involved in clopidogrel-induced gastric injury through p38 MAPK activation. *Toxicology*.

[48] Lv H., Zhu C., Liao Y. (2015). Tenuigenin ameliorates acute lung injury by inhibiting NF-*κ*B and MAPK signalling pathways. *Respiratory Physiology and Neurobiology*.

