# Supplementary material for: Agarwood Alcohol Extract Protects against Gastric Ulcer by Inhibiting Oxidation and Inflammation
Source: Evid Based Complement Alternat Med. 2021 Sep 18;2021:9944685. doi: 10.1155/2021/9944685 (PMC8464430; doi:10.1155/2021/9944685)
Supplement: Supplementary Materials — Supplemental Files1. Graphical abstract. Supplemental Files2. Highlights. Supplemental Files3. Chromatogram of chemical components of WTAAE. Supplemental Files4. Table 1 Chemical compositions and relative amounts of the WTAAE. [file 9944685.f1.zip › 9944685.f1/Supplemental Files2-Highlights.docx]

**Highlights:**

► Agarwood displayed anti-ulcer effect by inhibiting ulcer index and histological damage.

► Peroxide production and pro-inflammatory cytokine secretion decreased with treatment of agarwood extract.

► Mechanism of gastric ulcer protection associated with inhibiting oxidation and inflammation.
