# Supplementary material for: Agarwood Alcohol Extract Protects against Gastric Ulcer by Inhibiting Oxidation and Inflammation
Source: Evid Based Complement Alternat Med. 2021 Sep 18;2021:9944685. doi: 10.1155/2021/9944685 (PMC8464430; doi:10.1155/2021/9944685)
Supplement: Supplementary Materials — Supplemental Files1. Graphical abstract. Supplemental Files2. Highlights. Supplemental Files3. Chromatogram of chemical components of WTAAE. Supplemental Files4. Table 1 Chemical compositions and relative amounts of the WTAAE. [file 9944685.f1.zip › 9944685.f1/Supplemental Files4-The chemical compositions of WTAAE.docx]

Tab. 1 Chemical compositions and relative amounts of the WTAAE

| No. | Retention  Time (min) | Compounds | Relative Amoumt (%) |
| --- | --- | --- | --- |
| 1 | 7.514 | (1*R*,2*R*,3*S*,4*R*,5*R*)-2-[[(1,1-dimethylethoxy) carbonyl]amino]-4,6,6-trimethylbicyclo[3.1.1] heptane-3-carboxylic acid | 0.032 |
| 2 | 7.877 | Benzaldehyde | 0.155 |
| 3 | 21.215 | Benzylacetone | 0.116 |
| 4 | 47.521 | (*E*)-4-(Dimethylamino)-4′-methoxychalcone | 0.560 |
| 5 | 52.280 | Elemicin | 0.195 |
| 6 | 53.193 | *Cis*-(*α*)-santalol | 0.190 |
| 7 | 55.844 | Methoxycycloheptane | 0.154 |
| 8 | 56.705 | Hexadecane | 0.751 |
| 9 | 58.715 | (+)-*α*-Elemene | 0.218 |
| 10 | 58.987 | (-)-Hinesol | 0.082 |
| 11 | 59.472 | 1-Isopropyl-7-methyl-4-methylene-1,2,3,4,4*α*,5,6,8*α*-octahydronaphthalene | 0.262 |
| 12 | 59.888 | Rel-(1*E*,3*αR*,7*αS*)-1-ethylideneoctahydro-7*α*-methyl-1*H*-indene | 0.399 |
| 13 | 60.396 | 2-Isopropyl-5-methyl-9-methylenebicyclo[4.4.0]dec-1-ene | 1.246 |
| 14 | 61.430 | 1-Chlorooctadecane | 0.195 |
| 15 | 61.765 | Bicyclo[4.1.0]heptane-1-propanol,2,2,6-trimethyl-*α*-methylene-,1-acetate | 0.765 |
| 16 | 62.343 | 1-Benzyl-3,3-dimethyldiaziridine | 0.244 |
| 17 | 63.464 | 4-Methyl-2-cyclohexylphenol | 1.338 |
| 18 | 64.717 | Spiro[4.5]dec-6-en-8-one,2-[2-hydroxy-1-(hydroxymethyl)ethyl]-6,10-dimethyl-, [2*R*-[2*α*,5*α*(*S**)]]- | 0.097 |
| 19 | 65.410 | 3,9-Dioxa-6-thia-2,10-disilaundecane,2,2,10,10-tetramethyl-,6-oxide | 2.380 |
| 20 | 66.773 | 1-Heptadecene | 0.190 |
| 21 | 67.108 | Heptadecane | 0.463 |
| 22 | 67.715 | 3,6-Dimethyldecane | 0.463 |
| 23 | 69.598 | Hexadecahydropyrene | 0.312 |
| 24 | 69.835 | *o*-Tertbutyl phenol | 1.153 |
| 25 | 70.488 | (2*S*,3*S*,6*S*)-2-isopropenyl-6-isopropyl-3-methyl-3-vinylcyclohexanone | 0.365 |
| 26 | 71.626 | 2-Hexadecyloxyethanol | 0.223 |
| 27 | 72.007 | 3,5-Ditertbutyl-4-hydroxybenzaldehyde | 0.148 |
| 28 | 72.226 | 1,3-Dimethylene-2,2,5,5-tetramethylcyclohexane | 0.810 |
| 29 | 73.462 | 2-Hexyl-1-decanol | 0.211 |
| 30 | 73.740 | 2-(2-Hydroxyl-1-oxopropyl)-*α*-oxobenzeneacetic acid | 0.425 |
| 31 | 74.167 | 2,6,11-Trimethyl-dodecane | 0.401 |
| 32 | 74.843 | Eremophila-7(11),9-dien-8-one | 0.200 |
| 33 | 75.114 | 1,1,7-Trimethyl-4-methylenedec ahydro-1*H*-cyclopropa[*e*]azulene | 0.161 |
| 34 | 75.831 | Ledol | 0.312 |
| 35 | 77.154 | Octadecane | 0.429 |
| 36 | 77.587 | 4-Fluorobenzyl alcohol | 1.008 |
| 37 | 78.020 | 5-(2-Thienyl)-4-pyrimidinamine | 4.473 |
| 38 | 78.603 | (2,6,6-Trimethyl-2-hydroxycyclohexylidene)acetic acid lactone | 0.214 |
| 39 | 79.967 | 1,3,3-Trimethyl-2-[(1*Z*)-3-methyl-2-methylene-3-Buten-1-ylidene]cyclohexanol | 0.150 |
| 40 | 80.590 | Benzene, 1,2-diethyl-3,4-dimethyl | 0.508 |
| 41 | 81.515 | 1-Azaspiro[5.5]undecan-2-one,8-(acetyloxy)-7-butyl-,(6*α*,7*β*,8*α*)- | 3.065 |
| 42 | 82.959 | 4-Methyl-2-(1,1,3,3-tetramethylbutyl)phenol | 2.645 |
| 43 | 83.791 | 1,5,9,13-Tetradecatetraene | 0.404 |
| 44 | 86.124 | (+)-pyrethrolone | 0.317 |
| 45 | 86.812 | Nonadecane | 0.884 |
| 46 | 87.314 | - 7,8-Dihydro-4,7,7-trimethyl-2,5(1H,6H)-quinolinedione | 0.767 |
| 47 | 88.233 | Methyl eugenol | 0.536 |
| 48 | 88.700 | β-Elemene | 0.341 |
| 49 | 89.139 | (+)-Ledene | 2.576 |
| 50 | 89.925 | 4-(Octyloxy)benzaldehyde | 0.403 |
| 51 | 91.421 | Velleral | 1.806 |
| 52 | 91.710 | Dibutyl phthalate | 1.880 |
| 53 | 92.513 | 3,5-Dimethylaniline | 0.606 |
| 54 | 94.864 | (*Z*)-9-heptadecene-4,6-diyn-8-ol | 0.158 |
| 55 | 95.268 | 1*H*-pyrazole-3-carboxylic acid, 2,5-dihydro-5-oxo-2-phenyl-, ethyl ester | 0.120 |
| 56 | 95.343 | (-)-Culmorin | 0.054 |
| 57 | 96.019 | 4-[2-(5-Nitro-2-furanyl)ethenyl]-2-quinolinamine | 2.939 |
| 58 | 96.718 | 4,5-Epoxy-4,11,11-trimethyl-8-methylenebicyclo(7.2.0)undecane | 1.088 |
| 59 | 99.352 | 3,3,5-Trimethyl-5-phenylcyclohexanone | 0.670 |
| 60 | 99.843 | 2-Hydroxy-5-(3-methyl-2-butenyl)-4-(1-methylethenyl)-2,4,6-cycloheptatrien-1-one | 0.361 |
| 61 | 100.877 | 1-Methoxyl-2-(1-methyl-2-methylenecyclopentyl)benzene | 0.712 |
| 62 | 104.037 | 1,1,7-Trimethyl-4-methylenedec ahydro-1*H*-cyclopropa[e]azulene | 0.492 |
| 63 | 104.545 | Methyl-9-(*Z*)-octadecenoate | 0.105 |
| 64 | 108.496 | N-ethyl-4-(1-methylethyl)-N-(phenylmethyl)benzamide | 2.439 |
| 65 | 109.178 | (Ethenylsulfinyl)benzene | 0.069 |
| 66 | 112.205 | Cyclotridecane | 0.179 |
| 67 | 115.555 | 7-Hydroxy-3-(1,1-dimethylprop-2-enyl)coumarin | 0.164 |
| 68 | 116.774 | 2-(2-Phenylethyl)chromone | 3.357 |
| 69 | 117.010 | 9-(Methylaminomethyl)anthracene | 3.311 |
| 70 | 121.949 | 3-Hydroxy-2-methylpyrido[3,2-d]pyrimidin-4(3*H*)-one | 0.314 |
| 71 | 123.786 | 3,6-Dioxa-2,4,5,7-tetrasilaoctane, 2,2,4,4,5,5,7,7-octamethyl- | 2.338 |
| 72 | 125.057 | Octadecanohydrazide | 0.287 |
| 73 | 126.103 | [[1-(Phenylmethyl)-1*H*-indazol-3-yl]oxy]acetic acid | 0.240 |
| 74 | 127.749 | Dioctyl phthalate | 0.162 |
| 75 | 127.945 | Medicarpin | 1.026 |
| 76 | 128.702 | 6,7-Dimethoxyl-2-(2-phenylethyl) chromone | 7.074 |
| 77 | 129.695 | Pentoxifylline | 2.337 |
| 78 | 130.267 | 5,8-Dihydroxyl-2-(2- phenylethyl)chromone | 7.283 |
| 79 | 130.776 | 5-Hydroxyl-7-methoxyl-2-(2-phenylethyl) chromone | 1.351 |
| 80 | 132.976 | 6-Hydroxyl-2-(2-phenylethyl)chromone | 1.877 |
| 81 | 133.652 | (2*S*)-2-[bis(phenylmethyl)amino]-4-methylpentanal | 0.211 |
| 82 | 135.039 | Benzene,1,2,3,5-tetramethyl-4-(2-phenylethyl)- | 1.914 |
| 83 | 136.361 | 2-Ethylacridin | 0.067 |
| 84 | 137.545 | 5-Amino-N-methyl-N-[4-(1-pyrrolidinyl)-2-butyn-1-yl]pentanamide | 1.027 |
| 85 | 138.152 | 2-(Phenylmethoxy)benzeneethanamine | 1.134 |
| 86 | 138.458 | 3-[4-(Benzyloxy)phenyl]propionic acid | 0.170 |
| 87 | 138.753 | Methyl *p*-propylphenylether | 0.522 |
| 88 | 139.186 | 7-Methoxyl-2-(2-phenylethyl)chromone | 9.247 |
| 89 | 139.561 | 4-(4-Methoxyphenyl)-1-butanol | 2.069 |
| 90 | 139.943 | 1,4-Diisopropylbenzene | 1.728 |
| 91 | 141.277 | 2-Adamantanone azine | 0.193 |
| 92 | 143.582 | 7-Methoxyl-2-[2-(4'-hydroxylphenylethyl)] chromone | 0.478 |
| 93 | 144.483 | 2-Amino-4-(p-nitrophenyl)thiazole | 0.620 |
| 94 | 147.666 | 2-[2-(4'-Hydroxylphenylethyl)]chromone | 1.011 |
| 95 | 148.717 | 2-(4-Fluorophenyl)-6-methylquinoline-4-carboxylic acid | 0.268 |
| 96 | 150.872 | N-methyl-1-adamantaneacetamide | 0.166 |
| 97 | 152.581 | 4-Hydroxy-3,5-ditertbutyl-2,4-cyclohexadien-1-one | 0.233 |
| 98 | 152.697 | Benzo[*H*]quinoline,2,4-dimethyl- | 0.151 |
| 99 | 156.324 | 1-Methyl-2-phenylindole | 0.062 |
