# Supplementary material for: Agarwood Alcohol Extract Protects against Gastric Ulcer by Inhibiting Oxidation and Inflammation
Source: Evid Based Complement Alternat Med. 2021 Sep 18;2021:9944685. doi: 10.1155/2021/9944685 (PMC8464430; doi:10.1155/2021/9944685)
Supplement: Supplementary Materials — Supplemental Files1. Graphical abstract. Supplemental Files2. Highlights. Supplemental Files3. Chromatogram of chemical components of WTAAE. Supplemental Files4. Table 1 Chemical compositions and relative amounts of the WTAAE. [file 9944685.f1.zip › 9944685.f1/Supplementary Description_9944685.docx]

**Supplementary Description:**

Supplemental Files1—Graphical Abstract

Supplemental Files2—Highlights

Supplemental Files3— Chromatogram of chemical components of WTAAE were shown in the figure.

Supplemental Files4— Tab. 1 Chemical compositions and relative amounts of the WTAAE.
